# Validation of anti-aging drugs by treating age-related diseases

**DOI:** 10.18632/aging.100034

**Published:** 2009-03-28

**Authors:** Mikhail V. Blagosklonny

**Affiliations:** Cancer Center, Ordway Research Institute, Albany, NY 12208, USA

**Keywords:** anti-aging drugs, diseases, cancer, atherosclerosis, resveratrol, rapamycin, metformin

## Abstract

Humans die from age-related diseases, which are deadly manifestations of the aging process. In order to extend life span, an anti-aging drug must delay age-related diseases. All together age-related diseases are the best biomarker of aging. Once a drug is used for treatment of any one chronic disease, its effect against other diseases (atherosclerosis, cancer, prostate enlargement, osteoporosis, insulin resistance, Alzheimer's and Parkinson's diseases, age-related macular degeneration) may be evaluated in the same group of patients. If the group is large, then the anti-aging effect could be validated in a couple of years. Startlingly, retrospective analysis of clinical and preclinical data reveals four potential anti-aging modalities.

## Problem

The discovery of anti-aging drugs is no longer a fantasy. Numerous genes for aging and longevity have been identified in diverse organisms, revealing potential targets for potential anti-aging drugs. But how could potential anti-aging drug be introduced to humans? There are two problems. First, the effect of anti-aging agents on human aging may require almost a lifetime to determine [1]. Second, it is seemingly desirable to test anti-aging drugs in healthy individuals. However, all drugs have side effects. And, in healthy individuals, side effects would preclude clinical trials. How might these problems be solved? How could we validate anti-aging drugs in humans without life-long trials in healthy individuals?

## Solution

The solution includes two steps. First, we must find an indication for a drug to treat at least one chronic disease. Then this drug could be tested in humans, not as an anti-aging drug, but as therapy for a particular disease. In fact this approach has been suggested for introduction of activators of sirtuins to the clinic [2,3].

Second, we must find a biomarker of aging that absolutely predicts longevity. Then using this biomarker, the anti-aging effect could be evaluated in the same patients.

## Aging and age-related diseases

Aging can be defined as an increase in the probability of death. This is how the rate of aging can be measured. Humans die not from ‘healthy' aging but from age-related diseases. Healthy aging (a late onset of disease) is associated with longevity. For example, centenarians show significant delay in the onset of age-related diseases, including cardiovascular disease, type 2 diabetes, cancer and Alzheimer's disease. In other words, those who live longer are healthier and vice versa [4,5]. Since, by definition, all age-dependent diseases are connected with aging, these diseases are connected to each other. In fact, aging humans often suffer from many diseases simultaneously: diabetes, atherosclerosis, hypertension, macular degeneration, prostate enlargement and prostate cancer (in men) or breast cancer (in women), Alzheimer's disease and osteoarthritis. This is why elimination of one disease (e.g., cancer) will not radically extend maximal human lifespan. And as calculated, "the complete resolution of Alzheimer's disease would add about 19 days onto average life expectancy" [6]. But if a drug delays or stops all diseases, a person must live longer. Otherwise what would be the cause of death, if all causes were delayed? Since human longevity is limited by death from age-related diseases, a true anti-aging drug must delay age-related diseases. In other words, unless a drug delays age-related diseases, it will not extend lifespan. And vice versa, if a drug prevents age-related diseases, it must extend life span.

## Biomarker of organismal aging

Given that (a) an increase in the death rate is a measure of aging and (b) the death rate is determined diseases taken together, then we can conclude that the sum of all age-related diseases is the best biomarker of aging. Any one age-related disease is not a biomarker of aging because, in addition to aging, numerous factors contribute to the incidence of a particular disease. For example, smoking increases the risk for lung cancer but not for Parkinson's disease. Yet, aging is a risk factor for both diseases. And, even for lung cancer, aging is a bigger risk factor than is smoking. Aging is the biggest risk factor for all age-related diseases. Whether aging and disease have a common mechanism or whether aging simply increases vulnerability to diseases, in any case, the inhibition of aging will delay diseases, thus extending life span.

## Disease-specific drugs versus anti-aging agents

Slowing aging would delay all age-related diseases. If a drug is effective against one particular disease only, such a drug is not anti-aging. And current drugs are not anti-aging. For example, insulin compensates diabetes. Yet, insulin does not treat cancer. And vice versa chemotherapy may treat cancer but does not treat diabetes. So neither chemotherapy nor insulin is an anti-aging modality. Furthermore, both insulin and chemotherapy may accelerate aging.

## Metformin

The underlying cause of age-related type II diabetes is insulin resistance. Insulin treatment does not ‘treat' the cause, it just compensates for resistance. Unlike insulin, metformin, an oral anti-diabetic drug, restores insulin sensitivity in type diabetes type II. Remarkably, metformin decreases the incidence of breast cancer [7,8]. Also, metformin is considered for cancer treatment [9] and inhibits atherosclerosis in diabetic mice [10]. Metformin is used to induce ovulation in patients with polycystic ovary syndrome (PCOS). Six months of 1700 mg/d metformin treatment improved fertility in anovulatory PCOS women [11,12]. Given such effects on infertility, type II diabetes, cancer and atherosclerosis, it is plausible that metformin slows aging. In fact, it extends life span in rodents [13-15].

## Calorie restriction

Calorie restriction (CR) extends life span from yeast and worms to rodents and perhaps humans [16-18]. If we did not already know that CR slows aging, how might we figure that out based solely on clinical data? Unrestricted food consumption leads to obesity associated with diabetes, atherosclerosis, thrombosis, hypertension, cancer (especially breast, prostate and colon cancer), coronary heart disease, stroke, osteoporosis and Alzheimer's disease [19-25].  In other words, unrestricted eating in humans (ad libitum in rodents) accelerates most, if not all, diseases of aging. So we can conclude that CR delays all diseases of aging. This suggests that CR is an anti-aging modality. And it is known that CR extends life span in almost all organisms from yeast to mammals.

## From metformin and calorie restriction to rapamycin

**Figure 1.**
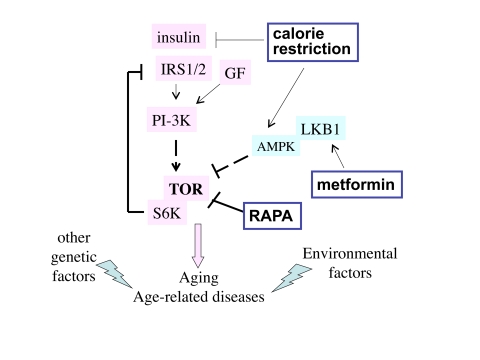
The TOR intracellular signaling pathway. Nutrients, GF (growth factors) and insulin activate the TOR pathway, which is involved in aging and age-related diseases. Other genetic factors and environmental factors (e.g., smoking) contribute to specific age-related diseases. Three potential anti-aging modalities (metformin, calorie restriction and rapamycin) all inhibit the TOR pathway.

Numerous factors including insulin, glucose and amino acids activate the nutrient-sensing TOR (target of rapamycin) pathway. When the TOR pathway is activated, it acts via S6K to deplete the insulin-receptor-substrate (IRS1/2), causing insulin resistance (Figure 1).  As shown in Figure 1, metformin indirectly (by activating AMPK) inhibits TOR and thereby restores insulin sensitivity [26].

CR decreases levels of nutrients and insulin and thus de-activates TOR (Figure 1). It is possible that the anti-aging effects of CR and metformin are due to inhibition of the TOR pathway. Like CR, rapamycin decreases size of fat cells and animal weight. When rats (15 weeks old) were either treated 1 mg/kg rapamycin 3 times per week for 12 weeks, rapamycin decreased their weight. Mean adipocyte diameter was decreased from 36 μm to 25 μm. At the end of the study, mean body weight in the rapamycin-treated rats was 356 g instead of 507 g, in spite of comparable food intake [27]. So rapamycin imitated CR. CR may also extend life span by activating sirtuins. Probably, sirtuins, AMPK and mTOR are linked in the common network [28].

Genetic inhibition of the TOR pathway slows down aging in diverse organisms, including yeast, worms, flies and mice [29-33]. If genetic inhibition of the TOR pathway slows aging, then rapamycin, a drug that inhibits TOR, must slow aging too. Once used for any indication, even unrelated to age-related diseases (such as renal transplantation, for instance), an anti-aging drug should slow down age-related diseases such as cancer, osteoporosis and atherosclerosis. Rapamycin is already used in renal transplant patients.

## Retrospective analysis of the clinical use of rapamycin

Rapamycin has been used in renal-transplant patients for several years. Since rapamycin was viewed as an immunossupressive drug (not as an anti-aging drug) it was expected that it would cause cancer.

Unexpectedly, it turned out that rapamycin prevented cancer, and even cured pre-existing cancer and Kaposi's sarcoma in renal transplant patients [34-44]. Furthermore, temsirolimus, an analog of rapamycin, has recently been approved for cancer therapy [45]. Also, everolimus, a TOR inhibitor, markedly delayed tumor development in transgenic mice that spontaneously develop ovarian carcinomas [46]. Would TOR inhibitors extend life span in transgenic mice? Since rapamycin delays cancer, it must prolong the life span of cancer-prone mice, who would otherwise die from cancer. Of course, humans die from a variety of age-related diseases, not from just one disease.  To prolong life span dramatically, rapamycin must delay most of them.

**Figure 2.**
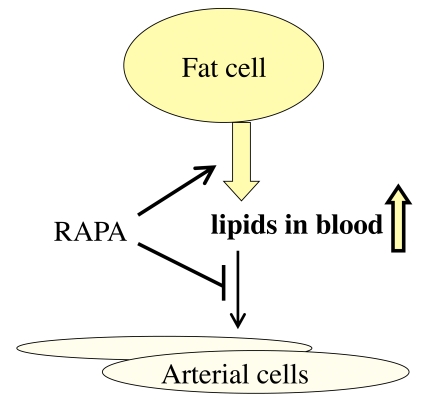
Re-interpretation of the hyperlipidemic side effect of rapamycin. Rapamycin activates adipose tissue lipase, thus mobilizing lipids from the fat tissue (lipolysis). This effect imitates starvation. Also, rapamycin inhibits lipoprotein lipase thus preventing utilization of lipids by the fat tissue and blocking  lipid uptake by the arterial wall. This results in increase in blood lipids.

In renal transplant patients, rapamycin increases blood lipoproteins [47]. This is considered to be a negative side effect. Yet, this results from mobilization of fat from the fat tissue (lipolysis) [48,49]. This is exactly what happens during starvation or calorie restriction (CR). And CR extends life span. Furthermore, rapamycin reduces the accumulation of cholesterol within the arterial wall [50,51]. Thus, lipolysis of fat tissue and decreased uptake of cholesterol by tissues both contribute to high levels of lipids in blood (Figure 2). Despite hypercholesterolemia, rapamycin prevents atherosclerosis in animals [52]. In animal models, systemic administration of rapamycin reduces neointimal thickening and slows the progression of atherosclerosis in apoE-deficient mice with elevated levels of cholesterol [53-55]. In patients with coronary atherosclerosis, oral rapamycin prevents re-stenosis after implantation of metal stents [56]. As a case report, it has been described that conversion to everolimus (an analog of rapamycin) resulted in decrease in blood pressure [57]. In kidney transplant patients, 2 years after transplantation, body-mass index was significantly lower in the rapamycin-based treatment arm compared to cyclosporine [27].

## Multiple indications for a single drug

If a drug is indicated to treat most age-related diseases, then this drug could be defined as an anti-aging drug. The probability that a non-anti-aging drug would have independent activities against all diseases is exceedingly low.

Rapamycin analogs are approved to treat certain cancers [45]. Based on preclinical data, rapamycin has been considered in such pathologies as obesity [58], atherosclerosis [53-55], cardiac hypertrophy [59-64], aortic aneurysm [65], osteoporosis [66-68], organ fibrosis (liver, renal, cardiac fibrosis) [64,69,70-75], neurodegeneration [76,77], Alzheimer's disease [78,79], Parkinson's disease [80-82], psoriasis [80], skin scars and keloids [83], multiple sclerosis [84], arthritis [85,86], and renal hypertrophy in diabetes [87].

## May rapamycin increase human life span?

In principle, life-extending effect of anti-aging drug might be limited by side effects. Although chronic administration of rapamycin is associated with some undesirable effects in transplant patients (see for references [88]), they might be avoided by administrating rapamycin in pulses (for example, once a week). For example, chronic administration of rapamycin impairs wound healing. In theory, a pulse treatment might rejuvenate wound-healing cells [88]. A single dose of rapamycin reverses insulin resistance, whereas chronic administration of rapamycin may precipitate diabetes in certain conditions. Clinical trials will be needed to determine benefits of pulse treatment with rapamycin. Alternatively, rapamycin can be combined with ‘complementary' drugs. Thus, hyperlipidemia caused by rapamycin may deteriorate insulin-resistance. Yet, hyperlipidemia caused by rapamycin can be controlled by lipid-lowering drugs. A combination of rapamycin with resveratrol may be especially intriguing.

## Resveratrol

Resveratrol, an activator of SIRT1 in mammals, extends life span in diverse species [89,90]. Resveratrol was shown to prevent cancer, atherosclerosis, neuro-degeneration and insulin-resistance (diabetes type II) [10,91-100]. Resveratrol also indirectly inhibits PI-3K/mTOR/S6K pathway [101-105]. SIRT1 and mTOR could be members of the same sirtuin/TOR network. The link between TOR and sirtuins has been suggested [28]. It is likely that TOR (pro-aging pathway) and sirtuins (anti-aging pathway) antagonize each other [106]. However, inhibition of the TOR pathway by resveratrol occurs at near-toxic concentrations [107].

The ability of resveratrol to extend life span may be limited by its toxicity at high doses due to off-target effects. Therefore, more selective activators of SIRT1 undergo clinical trials [3]. Importantly, these drugs will be developed to treat age-related diseases such as type 2 diabetes [3]. This is the only possible strategy for a drug to enter the clinic. But here is an additional aspect: this is the only practical way of how anti-aging effect can be evaluated too. Once used for treatment of diabetes, sirtuin activators might delay heart diseases, cancer, neurodegeneration and other age-related diseases in the same patients. And delaying of all diseases must extend life span, thus validating a drug as anti-aging.

## Conclusion

It was previously assumed that anti-aging drugs should be tested in healthy individuals. Ironically, the best biomarker of aging is the occurrence of age-related diseases. And this is how anti-aging drugs can be validated in the clinic (by showing that a putative anti-aging drug can prevent or delay the onset of all age-related diseases). Then such drugs could be approved for prevention of any particular age-related disease in healthy individuals. Thus, potential anti-aging drugs should be introduced to the clinical trials for therapy of a particular disease but be ultimately approved for prevention of all age-related diseases in healthy individuals. And this is synonymous to the approval of a drug as anti-aging.
